# Soil heterotrophic respiration assessment using minimally disturbed soil microcosm cores

**DOI:** 10.1016/j.mex.2018.07.014

**Published:** 2018-07-20

**Authors:** Louis-Pierre Comeau, Derrick Y.F. Lai, Jane Jinglan Cui, Jodie Hartill

**Affiliations:** a Department of Geography and Resource Management, Chinese University of Hong Kong, Shatin, Hong Kong; b Fredericton Research and Development Centre, Agriculture and Agri-Food Canada, Fredericton, NB E3B 4Z7, Canada; c Institute of Biological and Environmental Science, University of Aberdeen, 23 St Machar Drive, Aberdeen AB24 3UU, UK

**Keywords:** Soil respiration, Soil health assessment, Respiration by heterotroph microorganisms, Soil organic matter CO_2_ flux partitioning

## Abstract

Ex-situ measurement of soil respiration is usually done with highly disturbed samples that may confound the interpretation and extrapolation of results. We have developed a lab respiration assessment method that better simulates field conditions and allows efflux estimations based on soil surface area. First, intact soil cores are extracted in the field and transferred to the lab. Next, soil moisture content and bulk density are assessed in each soil core. Immediately following this the soil cores are gently broken, pooled per treatment (or plot) and the root systems removed. Subsequently the field moist, non-sieved soils are repacked into microcosm cores at their respective bulk densities. Moisture content in the microcosms is adjusted to desired levels by adding drops of deionized water or by air drying for several hours. After moisture adjustment, the cores are pre-incubated at 25 °C for two weeks. Afterwards, the microcosms are further incubated in the dark at the desired temperatures in airtight containers. At incubation times of 0, 48 and 96 h, 20 ml of gas sample is collected from each container via the septum, and then injected into pre-evacuated exetainers for CO_2_ determination using a gas chromatograph or an infrared gas analyzer. Finally, soil efflux is estimated based on the rate of linear CO_2_ increase in the container headspace. One of the advantages of this method is that results can be presented per unit of mass (e.g. mg CO_2_-C g soil^−1^ day^−1^) or area (e.g. g CO_2_-C m^2^ day^−1^). These soil microcosms can also be used to simultaneously assess emissions of CH_4_ and N_2_O during incubations.

This new method uses:

• Small intact soil cores collected in the field.
• Soil microcosms.
• Efflux calculated per unit of area.

## Graphical abstract

**Figure:**
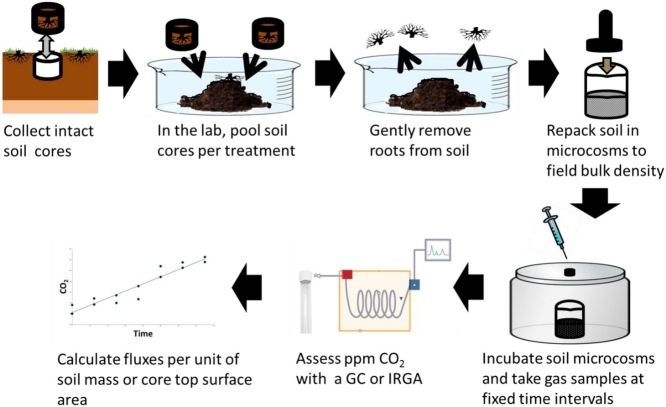

**Specifications Table**

**Table:** 

Subject area	*Agricultural and Biological Sciences*
More specific subject area	*Soil and Environmental Science*
Method name	*Soil respiration*
Name and reference of original method	*Soil respiration with intact soil cores: Comeau L-P., Carbon dioxide fluxes and soil organic matter characteristics on an intact peat swamp forest, a drained and logged forest on peat, and a peatland oil palm plantation in Jambi, Sumatra, Indonesia. Ph.D. thesis. University of Aberdeen, July 2016.*
Resource availability	

## Method details

Soil heterotrophic respiration (Rh) measures the metabolic activity of the soil microbial communities [1,2]. This assessment is particularly important for soil health evaluation and soil carbon balance assessment [3,4]. Ex-situ determination of soil respiration usually involves rewetting dried and sieved samples of soil in the laboratory; a procedure that potentially causes significant disturbance to the soil structure, and thus affecting the dynamics of soil microorganisms [5]. Consequentially, interpretation and extrapolation of the results are challenging [6,7]. We have developed and tested a lab incubation method that is easy to implement and better simulates field conditions [8].

First, in the field, undisturbed soil cores with a volume of 98 cm^3^ (inner diameter 5 cm, height 5 cm) are collected vertically from the upper part of the soil profile (0–5 cm) (details about soil cores extraction with bulk density rings can be found in “Soil Sampling and Methods of Analysis” [9]). Between 4 and 10 soil cores are collected per treatment (or per field plot) and immediately stored in coolers with ice packs. The number of soil cores collected should be based on plot size and intrinsic spatial heterogeneity. Subsequently, in the lab, the soil cores are weighted and the soil is carefully retrieved from the cores. Subsamples (one per core) of 20 g are collected and oven dried at 105 °C to assess the soil bulk density and moisture content. Soil cores are then pooled per treatment and root systems are removed taking special care not to destroy the small aggregates. Root removal is essential when working in agricultural fields that have mature plants growing in the soil or with forest soil. Omitting root removal could create abnormal CO_2_ pulses from high decomposition rates of the freshly excised roots [10,11]. After root removal, the soil is repacked into 51 cm^3^ microcosm cores (see method below to build microcosm tubes) at their respective bulk density. The amount of soil needed in the microcosms can be calculated with Eq. (1).

(1) $$Sn=Vo.X.Db.X.(.\frac{Gv}{100}.+1)$$

*Sn*, fresh soil required to fill microcosm (g); *Vo*, volume of the microcosm (cm^3^); *Db*, soil bulk density (g cm^−3^); *Gv*, soil gravimetric moisture content (%).

The mass of fresh soil needs to be calculated based on values derived from the individual field plot with specific values of bulk density and soil moisture content. To optimize the bulk density uniformity, microcosms are created in a two separate layers: 1) half of the soil is repacked into the bottom half (2.5 cm thick) of the microcosm core, while 2) remaining soil is repacked into the microcosm top half (2.5 cm thick). After the soil has been repacked into the microcosm tubes, soil moisture content is adjusted to desired levels by adding drops of deionized water or by air drying for several hours. The amount of water needed in the microcosms to reach desired moisture level can be calculated with Eq. (2).

(2) $$Wa=Wm+Wf+(Vo.X.Db.X.\left(.\frac{Dw}{100}.+1\right))-.Wm+Wf+(Vo.X.Db.X.\left(.\frac{Cw}{100}.+1\right))$$

*Wa*, water needed for desired soil moisture content level (ml); *Wm*, weight of the empty microcosm tube (g); *Wf*, weight of the moist paper filter at the bottom of the microcosm (g); *Db,* soil bulk density (g cm^−3^); *Vo*, volume microcosm (cm^3^); *Dw*, desired soil gravimetric moisture content level (%); *Cw*, current soil gravimetric moisture content (%).

For standard heterotrophic respiration assessment, a soil moisture content of 50% water holding capacity (WHC) is advised. This moisture level is recommended to ensure staying below the critical threshold for denitrification during the incubations [[12], [13], [14]]. Refer to the section entitled: “determination of WHC” below to determine and adjust this parameter. After moisture adjustment, each individual microcosm is covered with Parafilm^®^ (to prevent evaporation) and pre-incubated at 25 °C for two weeks to allow the soil to stabilize in a hermetically sealed plastic container. We recommend the minimum volume of the containers to be 2.5 l to ensure constant aerobic conditions throughout the incubation period. In addition, the containers should be at least 15 cm high to make sure the needle does not hit the microcosm when extracting the gas. After the pre-incubation, any soil microcosms with germinated seeds in them must be discarded. Also, after the pre-incubation, microcosms should be weighed again to ensure that soil moisture has not altered during the pre-incubation period. For standard Rh assessment, an incubation temperature of 25 °C is advised to warrant high rates of microbial activity [15]. Alternatively, several levels of temperature and soil moisture can be used to simulate field conditions in the different seasons or climate scenarios. From all incubation containers, gas samples are collected (20 ml) with an air-tight syringe at 0, 48, and 96 h after container closure using a septum. To prevent stratification of the gases during the sampling period the headspace air in the chambers is thoroughly mixed. Specifically, immediately before gas sampling, the air inside the containers is quickly pumped in and out with a vacuumed 60 ml syringe in a close circuit for 15 s to generate an air flow and homogenize the gas. Alternatively some studies have used an internal micro-fan in the chamber headspace to mix the air [16]. The CO_2_ concentrations are analyzed within 48 h of sample collection using a gas chromatograph (GC). Also, the samples can be injected into an infrared gas analyzer (IRGA) and the CO_2_ (ppm) recorded.

## Carbon efflux calculation

The rate of CO_2_ efflux from each microcosm is determined by linear regression of CO_2_ concentrations against time. Microcosm fluxes with a coefficient of determination r^2^ < 98 or with a Pearson's chi-squared p-value >0.05 should be discarded. The ideal gas law is used to convert CO_2_ in ppm, to carbon mass. The conversion factor of ppm CO_2_ to μg CO_2_-C m^−3^ is calculated with Eq. (3).

(3) $$Cf=\frac{P.X.Mwc.X.1000}{R.X.T}$$

*Cf,* conversion factor of ppm CO_2_ to μg CO_2_-C m^3^; *P*, air pressure (kPa); Mwc, molar mass of carbon (12); *R*, gas constant (8.314); *T*, incubation air temperature (K).

Finally, the Rh efflux can be computed either on mass or area basis. The CO_2_-C per unit of dry soil mass can be calculated following Eq. (4).

(4) $$Fmass=.\frac{\frac{\Delta .(Ppm.X.Cf)}{\Delta t}.X.Hs}{Vo.X.Db.}$$

*Fmass,* linear gas efflux in incubation container on soil mass basis (μg CO_2_-C g^−1^ soil h^−1^)*; Ppm,* CO_2_ concentration measured with the IRGA or GC (ppm); *Cf*, conversion factor of ppm CO_2_ to μg CO_2_-C m^-3^; *Δt,* incubation time (hours); *Δ(Ppm X CF),* change in gas concentration during incubation period; Hs, headspace in the incubation container (m^3^); *Db,* soil bulk density (g cm^-3^); *Vo*, volume of the microcosm (cm^3^).

The CO_2_-C per unit of area can be calculated following Eq. (5).

(5) $$Farea=.\frac{\frac{\Delta .(Ppm.X.Cf)}{\Delta t}.X.Hs.X.10^{-6}}{Area}$$

*Farea,* linear gas efflux in incubation container on soil mass basis (g CO_2_-C m^2^ h^−1^)*; Ppm,* CO_2_ concentration measured with the IRGA or GC (ppm); *Cf*, conversion factor of ppm CO_2_ to μg CO_2_-C m^-3^; Δ*t,* incubation time (hours); Δ*(Ppm X CF),* change in gas concentration during incubation period; Hs, headspace in the incubation containers (m^3^); *10^-6^*, conversion factor from μg to g; *Area*, area of the microcosm surface (m^2^).

## Method to build the microcosm tubes

**Fig. 1:**
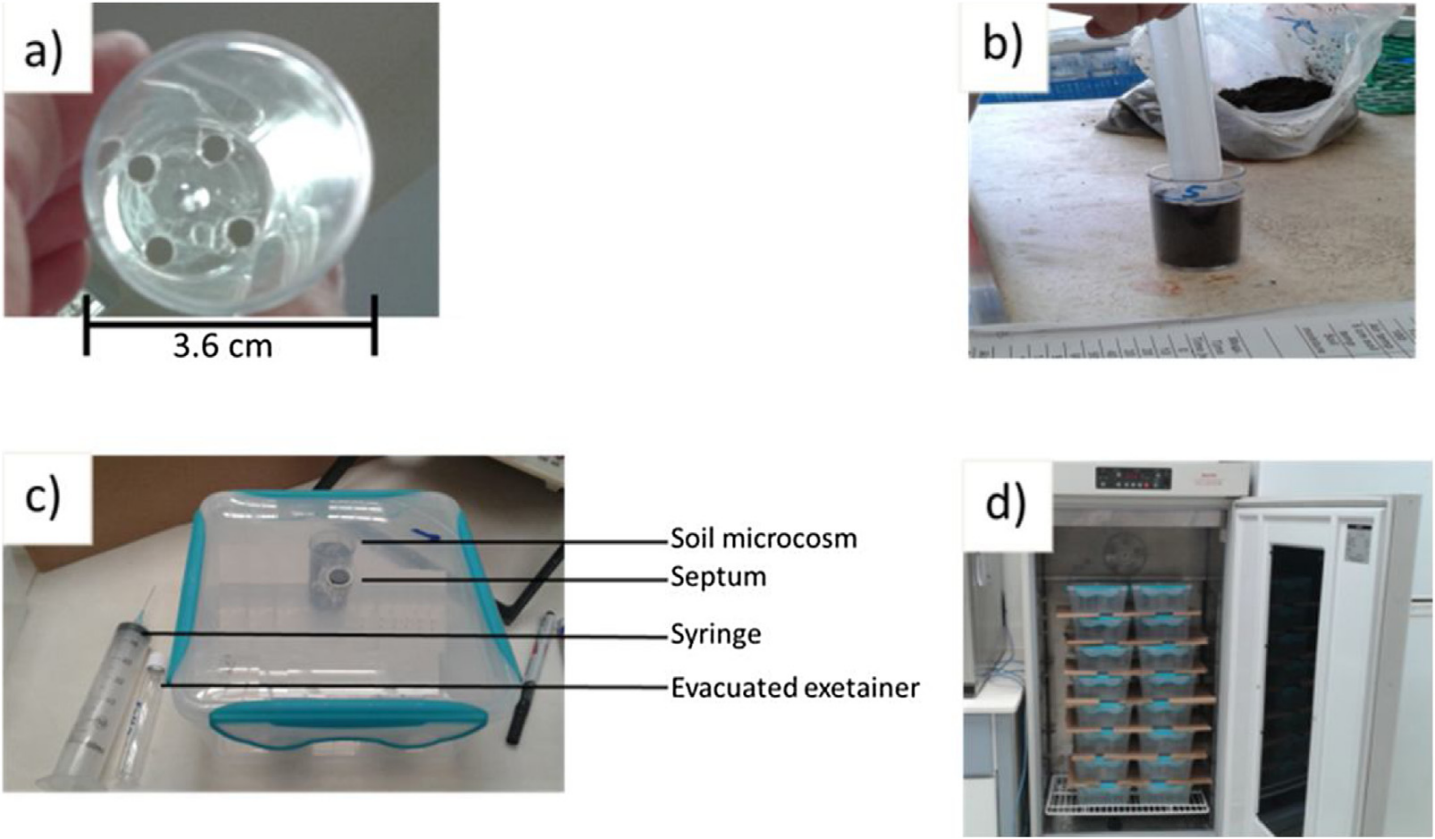
a) Empty microcosm tube with 4 holes at the bottom; b) soil repacked into 51 cm^3^ microcosm core; c) 3 l airtight incubation container with soil microcosm inside; d) incubation containers stacked in the incubator.

The microcosm tubes are constructed from transparent polystyrene vials (60 ml; 36 mm inside diameter). Using a sharp 0.8 mm auger drill bit, four holes are placed evenly through the bottom of the vials (Fig. 1a). The vials/microcosm tubes are labeled and their individual weight recorded. Laboratory filter paper (pore size 20–25 μm) is cut into disks that will fit into the inside bottom of the microcosm tubes. Three drops of deionized water is applied to each disk with excess water gently shaken from each one by hand. Disks should then be weighed and placed inside at the bottom of the microcosm. On the outside of each microcosm lines are drawn to indicate 2.5 cm and 5 cm from the base of the tube (Fig. 1b). The microcosms are at this point ready for the soil repacking process with the lines used as area boundaries for the bulk density adjustment.

## Method to assess water holding capacity determination with the microcosms

Soil WHC is determined for each plot. First, the fresh soil is softly compacted to its field bulk density value in the 51 ml microcosms in the same manner as the soil for the incubation described above. Second, the soil microcosms are saturated with water ponding and the top of the microcosm tubes are covered with Parafilm^®^ to prevent evaporation. Third, the soil microcosms are placed on a 2 mm sieve to drain the excess water through the bottom of the microcosm in the dark at 25 °C for 10 h without external pressure being applied. Fourth, the soil microcosms are weighed and the WHC is calculated using Eq. (6).

(6) $$WHC=\frac{WSM.-.Wm.-.Wf-.(Vo.X.Db)}{Vo.X.Db.}$$

*WHC,* water holding capacity (g H_2_O g soil^−1^); *WSM*, weight of water saturated microcosm (g); *Wm*, weight of empty microcosm tube (g); *Wf*, weight of moist paper filter at the bottom of the microcosm (g); *Dd,* soil bulk density (g cm^-3^); *Vo*, volume of the microcosm (cm^3^).

For standard incubation, the soil moisture content is adjusted to 50% WHC. The soil gravimetric water content equivalent to 50% WHC can be calculated with Eq. (7).

(7) $$Dw50=\frac{\left(.WSM.-.Wm.-.Wf-.\left(Vo.X.Db\right)\right).X.0.50}{Vo.}.X.100$$

*Dw50,* soil gravimetric water content at 50% WHC (%); *WSM*, weight of the water saturated microcosm (g); *Wm*, weight of the empty microcosm tube (g); *Wf*, weight of the moist paper filter at the bottom of the microcosm (g); *Db,* soil bulk density (g cm^−3^); *Vo*, volume of the microcosm (cm^3^).

## Verifying method validity

To test the effectiveness of the minimally disturbed soil microcosm cores to assess soil heterotrophic respiration, we performed incubations on a set of samples with different temperature and soil moisture levels. Briefly, four control plots were selected at the Tai Po Kau Nature Reserve Research Station in Hong Kong [17]. Four 98 cm^3^ (inner diameter 5 cm, height 5 cm) intact soil cores were extracted in each plot. The procedures described above were followed. The incubations were conducted in a factorial design with 4 WHC (30, 48, 66 and 84%) and 4 temperatures (14 °C, 20 °C, 26 °C and 32 °C). The CO_2_ concentrations were analyzed with a gas chromatograph (GC system 7890 A, Agilent Technologies) equipped with a flame ionization detector and an electron capture detector to quantify CO_2_. The calculated carbon effluxes were plotted with a Gaussian 3D regression fitted curve following Eq. (8) using SigmaPlot version 10.0 (Systat Software, San Jose, CA).

(8) $$f\left(x,y\right)=a.\times \exp\left[-0.5.\times {\left(\frac{x-xo}{b}\right)}^{2}+{\left(\frac{y-yo}{c}\right)}^{2}\right].$$

*a*, *b* and *c* are constant coefficients; *x* is the soil temperature (°C); *y* is the soil moisture (%); *x0* is the average temperature; *y0* is the average soil moisture.

**Fig. 2:**
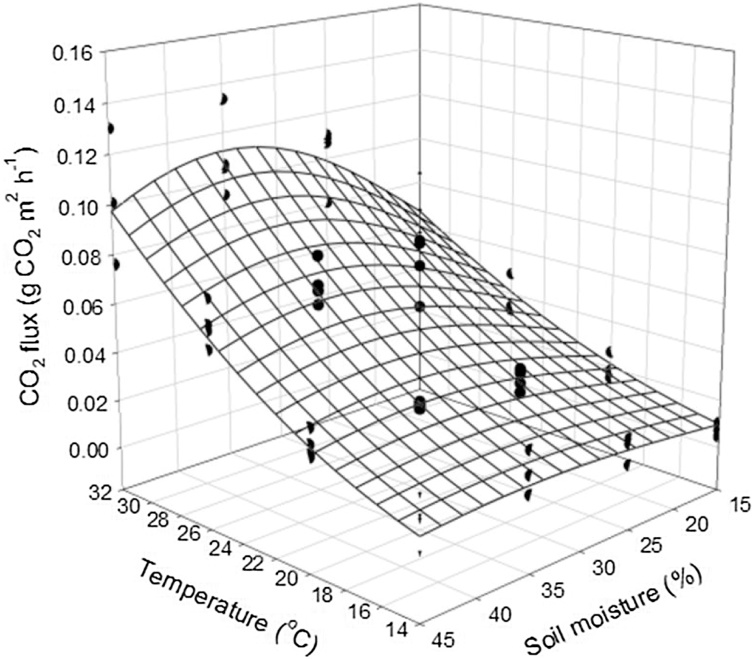
Relationships between temperature, moisture, and CO_2_ efflux determined from lab incubation data.

The resultant pattern produced (Fig. 2) was comparable to field measurements made in this location [17].

## Additional information

The volume of the microcosms can be modified to better simulate field conditions if needed. For example, if a very large amount of organic matter is present in the soil profile up to 8 cm depth then the microcosm should be built to 8 cm high. To accommodate this, it is critical that the height of the soil microcosms is equal to the height of the intact soil cores extracted in the field. It is also important that the soil is repacked in the microcosms in increments of 2.5 cm to ensure a homogeneous bulk density. Also, when working in non-vegetated agricultural fields (i.e. soil without live roots) the collected intact soil cores can be directly placed into the container after gravimetric moisture adjustment [18] or sealed headspace chambers can be placed on the top of larger microcosms [19]. In order to simultaneously assess the emissions of CH_4_ and N_2_O from the microcosm incubations, the molar mass of carbon should be replaced by the molar mass of these two molecules, respectively, in Eq. (3). This paper does not specify the manufacture and model of the instrument to measure CO_2_ concentration in the containers. The responsibility is given to the users to operate with precise and accurate equipment. The users are also responsible to make sure the vials and incubation containers are reliable, airtight and with sufficient headspace to ensure aerobic incubations.

## Conflict of interest

The authors declare that there are no conflicts of interest.
